# Open questions: how does *Wolbachia* do what it does?

**DOI:** 10.1186/s12915-016-0312-z

**Published:** 2016-10-19

**Authors:** Francis M. Jiggins

**Affiliations:** Department of Genetics, Downing Street, Cambridge, CB23 8AF UK

## Abstract

A common symbiont of insects, the bacterium *Wolbachia* has been implicated in phenomena as diverse as sex determination, pathogen defence and speciation and is being used in public health programs to prevent mosquitoes transmitting disease. Despite decades of research, we know remarkably little about how it exerts its effects.

## Comment

Thirteen years ago I was frustrated with my research and decided to change direction. I had been working on a bacterial symbiont called *Wolbachia* that is thought to infect over half of arthropod species—that’s over a million species [1]. As an evolutionary biologist I was fascinated by an organism that manipulated the reproduction of its hosts in bizarre ways to enhance its own transmission. As a geneticist, however, I was left frustrated. The bacterium could not be cultured or manipulated, so despite its being studied by hundreds of researchers, only the most rudimentary details were known about how it exerts its effects. I decided the time had come to move on and study interactions between *Drosophila* and viruses, so it was much to my surprise when five years later two studies reported that *Wolbachia* protects *Drosophila* against RNA viruses [2, 3]. Returning to the field, I found that the importance of *Wolbachia* in the biology of insects and other arthropods was more apparent than ever. The bacterium has been implicated in phenomena as diverse as speciation [4], the evolution of sex determination mechanisms [5] and the synthesis of essential vitamins [6]. Within a few years of its antiviral effects being reported, *Wolbachia-*infected mosquitoes were being released to prevent the transmission of dengue virus [7]. However, the mechanisms underlying these effects remain poorly understood.

The first description of a phenotypic effect of *Wolbachia* on its hosts came in 1971, when Yen and Barr [8] linked the bacterium to a phenomenon known as cytoplasmic incompatibility (CI). *Wolbachia* is transmitted from infected females to their offspring through eggs and CI allows it to rapidly spread through populations. The bacterium modifies the sperm of infected males during spermatogenesis so that the paternal chromosomes condense when an egg is fertilised, which typically kills the developing embryo [9]. However, if the egg is infected with the same strain of *Wolbachia* as was found in the male insect, this chromosomal mark is ‘rescued’ and development proceeds normally. At the population level, the selective killing of *Wolbachia-*free zygotes causes the frequency of infected individuals to increase. Despite detailed descriptions of how CI disrupts the cell cycle and paternal chromosomes [9], the molecular basis for how paternal chromosomes are marked and then rescued remains unknown. Advances in epigenetics and chromosome biology make this a timely moment to return to this question.

**Fig. 1:**
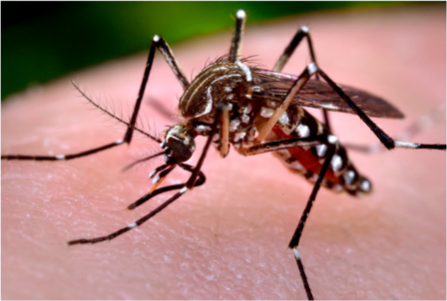
A female *Aedes aegypti* mosquito taking a blood meal from her human host. *Aedes aegypti* mosquitoes infected with *Wolbachia* are being released in Brazil, Colombia, Indonesia, Vietnam and Australia to prevent the transmission of dengue and Zika viruses. Photo credit: James Gathany

Thirty-seven years after *Wolbachia* was linked to CI, it was found that some strains make insects resistant to viruses [2, 3]. The combination of the two traits immediately suggested an application—if the symbiont were transferred to mosquitoes, CI would cause it to spread through populations where it could prevent the transmission of viral disease. Just three years later promising results were reported from trials in Australian populations of the mosquito *Aedes aegypti* [7] (Fig. 1) and much larger releases of *Wolbachia-*infected mosquitoes are now underway in areas where dengue virus is endemic. The use of *Wolbachia* in large public health programs makes it important to understand how it affects viral replication, as this may allow us to improve the efficiency of these efforts or predict undesirable outcomes. The antiviral effects of *Wolbachia* do not rely on antiviral RNA interference [10], which is the main immune defence of insects against viruses, and studies have implicated phenomena as diverse as microRNAs, reactive oxygen species and (perhaps most promisingly) competition for lipids in the trait [11–13]. However, it remains unclear whether different processes may account for the breadth of *Wolbachia*’s antiviral effects, which operate against a broad range of viruses in a diverse range of insects. The exact mechanism by which *Wolbachia* prevents viral replication remains unknown.

**Fig. 2:**
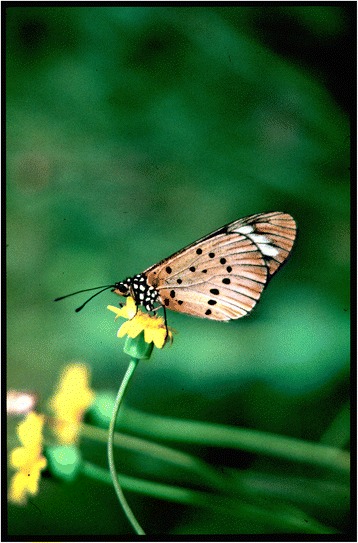
The butterfly *Acraea encedon* is a victim of *Wolbachia* sex ratio distortion. In populations of *A. encedon* over 99 % of females are infected by a strain of *Wolbachia* that kills their sons, leaving most females unable to find a mate. Photo credit: Roger Jiggins

Another common effect of *Wolbachia* is to distort sex ratios. Because the bacterium is only transmitted by infected females, it has evolved a diversity of ways to favour the production of daughters over sons. This includes killing sons, making infected individuals reproduce by parthenogenesis and feminising genetic males so they develop as females. In some species, such as the butterfly *Acraea encedon*, this can result in highly female-biased population sex ratios (Fig. 2). In some cases key mechanistic details of these processes have been known for many years. For example, parthenogenesis-inducing *Wolbachia* strains are frequently found in the Hymenoptera (bees and wasps), where haploid zygotes develop into males and diploids develop as females. This haplodiploid mechanism of sex determination is exploited by *Wolbachia*, which prevents the first cell division after the chromosomes have been replicated, converting a haploid male zygote into a diploid female zygote [5]. In woodlice, genetic males develop as females when *Wolbachia* disrupts a gland that produces a hormone required for male development [5]. However, the molecular basis of these manipulations remains unknown. Perhaps the best-understood sex ratio distorting strain comes from *Ostrinia* moths, where *Wolbachia* kills males by preventing the expression of *Masc*, which encodes a protein early in the sex determination pathway that is required for males to develop [14]. Even in this case, however, the mechanism by which *Wolbachia* has this effect remains elusive.

Some 45 years after the seminal work of Yenn and Barr, the interest of the research community in *Wolbachia* has never been greater. The field has gone from being an esoteric example of evolution to underpinning many aspects of insect biology and even being the basis of a major public health program. This research effort has yielded tantalising glimpses into the mechanisms by which *Wolbachia* alters the biology of its hosts, but progress has been slow. Critically, the bacterial factors that interact with the host have remained largely elusive. The challenge for the future is to answer these questions in a system where there are few tools for manipulating the bacterium. These limitations can be partly offset by the fact that *Wolbachia* infects model organisms such as *Drosophila* and new technologies and ‘omics approaches are easily applied to other species. Progress is likely to yield new insights into insect genetics and evolution. From the perspective of *Wolbachia*, it will reveal how this symbiont has evolved such a diverse array of phenotypes and whether common pathways underpin apparently disparate traits.
